# Programming of Obesity and Comorbidities in the Progeny: Lessons from a Model of Diet-Induced Obese Parents

**DOI:** 10.1371/journal.pone.0124737

**Published:** 2015-04-16

**Authors:** Fernanda Ornellas, Vanessa Souza-Mello, Carlos Alberto Mandarim-de-Lacerda, Marcia Barbosa Aguila

**Affiliations:** Laboratory of Morphometry, Metabolism, and Cardiovascular Disease, Biomedical Center, Institute of Biology, State University of Rio de Janeiro, Rio de Janeiro, Brazil; University of Cordoba, SPAIN

## Abstract

**Aim:**

To determine the impact of paternal obesity, maternal obesity or the combination of two obese parents on markers of adult offspring metabolism, with a focus on body mass (BM), lipid and carbohydrate, components of lipogenesis and beta-oxidation in the liver, sex dimorphism in the offspring that received a SC diet during the postnatal period.

**Materials and Methods:**

Male and female C57BL/6 mice were fed a high-fat diet (HF; 49% lipids) or standard chow (SC; 17% lipids) for 8 weeks before mating until lactation. The offspring were labeled according to sex, maternal diet (first letters), paternal diet (second letters), and received a SCdiet until 12-weeks of age when they were sacrificed. BM, eating behavior, glucose tolerance, plasma analysis, gene and protein expression of the components of lipogenesis and beta-oxidation in the liver of offspring were evaluated.

**Results:**

HF diet-fed mothers and fathers were overweight, hyperglycemic and glucose intolerant and had a deteriorating lipid profile. The adult male and female offspring of HF-mothers were overweight, with an increased adiposity index, hyperphagic, had an impaired glucose metabolism, increased total cholesterol and triacylglycerol levels, increased lipogenesis concomitant with decreased beta-oxidation resulting in liver steatosis. The male and female offspring of HF-father had impaired glucose metabolism, exacerbated lipogenesis without influencing beta-oxidation and enhanced hepatic steatosis. These findings are independent of BM. Male and female offspring of a mother and father that received a HF diet demonstrated these effects most prominently in adult life.

**Conclusion:**

Paternal obesity leads to alterations in glucose metabolism, increase in components of lipogenesis and liver steatosis. In contrast, maternal obesity leads to overweight and changes in the metabolic profile and liver resulting from activation of hepatic lipogenesis with impaired beta-oxidation. When both parents are obese, the effects observed in the male and female offspring are exacerbated.

## Introduction

According to the World Health Organization, in 2008, over half a billion adults were obese worldwide. In developed countries like the United States, medical costs related to obesity reached the milestone of $ 147 billion in the same period [1], with estimates exceed the range of 957 billion dollars in 2030 [2]. However, the increase in the prevalence of obesity has not happened only in developing countries, but also in developing countries such as Brazil [3], featuring a major public health problem.

The literature on the subject is vast and the results suggest that the risk of the development of obesity and metabolic syndrome in adulthood can be influenced by the initial period of life, especially through inadequate nutrition available to the fetus and/or newborn [4].

Studies in rodents have shown that the change in the mother's gestational metabolism can affect placental and embryonic development, culminating in increased inflow of nutrients via the placenta to the fetus, a fact that will influence their growth and result in irreversible adjustments in the structure and function of organs as the liver [5,6].

Decreased hepatic beta-oxidation rates and very low-density lipoprotein secretion coupled with increased *de novo* lipogenesis are usually associated with fat droplets accumulation within the liver of obese mothers’ offspring [7,8], characterizing nonalcoholic fatty liver disease (NAFLD). Excessive body mass and insulin resistance are often reported in this progeny, and both conditions are suggested to trigger and/or maximize NAFLD [9].

Given the multifactorial origin of obesity and that eating patterns are usually shared by individuals from the same family [10], consideration should also be given to the role that paternal obesity exerts upon the offspring. Although most of epidemiological and experimental investigations have focused maternal influence on the health of offspring, recent experiments conducted with rodents also demonstrated the paternal participation in metabolic programming of the adult offspring, affecting the glucose-insulin homeostasis and the lifespan of pancreatic islets in the daughters [11,12]. These impairments could be also transmitted to other generations [13]. Additionally, an investigation performed with parental diabetes among subjects of the population-based Framingham Offspring Study suggested that fathers may transmit unique paternal genetic characteristics of similar strength to maternal environmental factors, resulting in equivalent risk ratios for type 2 diabetes [14]. However, these findings are still scarce and controversial, indicating the need for further studies, especially comparing the effect of paternal and maternal nutritional status in the development of future diseases in descendants as well as possible interactions between parental nutritional statuses.

We aimed to determine the impact of paternal obesity, maternal obesity or a combination of two obese parents on markers of adult offspring metabolic syndrome, focusing on body mass, metabolism of lipid and carbohydrate, components of lipogenesis and beta-oxidation signaling pathways in the liver of male and female adult offspring that received a SC diet in the postnatal period.

## Materials and Methods

### Animals and diet

We used virgin male and female C57BL/6 mice (4-weeks old) in this study. The animals were maintained in an environment with a 12 h light/dark cycle, controlled temperature (21±2°C) and humidity (60±10%). Animal care and procedures were performed according to conventional guidelines for experimentation with animals (National Institutes of Health Publication No. 85–23 revised, 1996). The Ethics Committee for the use of animals of the State University of Rio de Janeiro (Comissão de Ética para o Cuidado e Uso de AnimaisExperimentais) approved all experimental procedures under the protocol number 070/2012.

The male mice were randomly divided into two groups (n = 30 per group) and were fed *ad libitum* a high-fat diet (HF, designated as group HF father, HF-Fa) or a standard rodent chow (SC, designated as group SC father, SC-Fa). Likewise, the female mice were randomly divided into two groups (n = 30 per group) and were fed *ad libitum* a high-fat diet (HF, designated as group HF mother, HF-Mo) or a standard rodent chow (SC, designated as group SC mother, SC-Mo).

In relation to experimental diets, the HF diet had 32% of the total energy from carbohydrates, 19% of the total energy from protein and 49% of the total energy from lipids. In the SC, 64% of the total energy was from carbohydrates, 19% of the total energy was from protein, and 17% of the total energy was from lipids. The energy availability of the diet was 20.7 kJ/g for the HF diet and 16.5 kJ/g for the SC diet. The diets were formulated with purified ingredients. The content of vitamins and minerals was identical for both experimental diets and followed the standards recommended by the American Institute of Nutrition for rodent diets to support growth during pregnancy, lactation and post-weaning periods of life (AIN-93G) [15]. Table 1 details the macronutrient composition and energy distribution in the HF and SC diet. The experimental diets were manufactured by PragSolucoes (Jau, SP, Brazil). The father and mother SC- and HF-fed mice received their respective experimental diets during the pre-mating period (from 4-weeks old until12-weeks old), and in the case of dams, the animals received the experimental diets during the gestation and lactation periods.

**Table 1:** Composition and energy content of standard chow (SC) and high-fat (HF) diet (AIN 93G based diets).

Nutrients (g/kg)	SC	HF
Casein	190.00	230.00
Corn starch	539.486	299.472
Sucrose	100.00	100.00
Soybean oil	70.00	70.00
Lard	-	200.00
Fiber	50.00	50.00
Vitamin Mixture (mg)	10.00	10.00
Mineral Mixture (mg)	35.00	35.00
Cysteine	3.00	3.00
Choline	2.50	2.50
Antioxidants	0.014	0.028
Total mass (g)	1,000.00	1,000.00
Energy (kJ/kg)	16,590.00	20,790.00
Carbohydrates (% Energy)	64	32
Protein (% Energy)	19	19
Lipids (% Energy)	17	49

**Fig 1:**
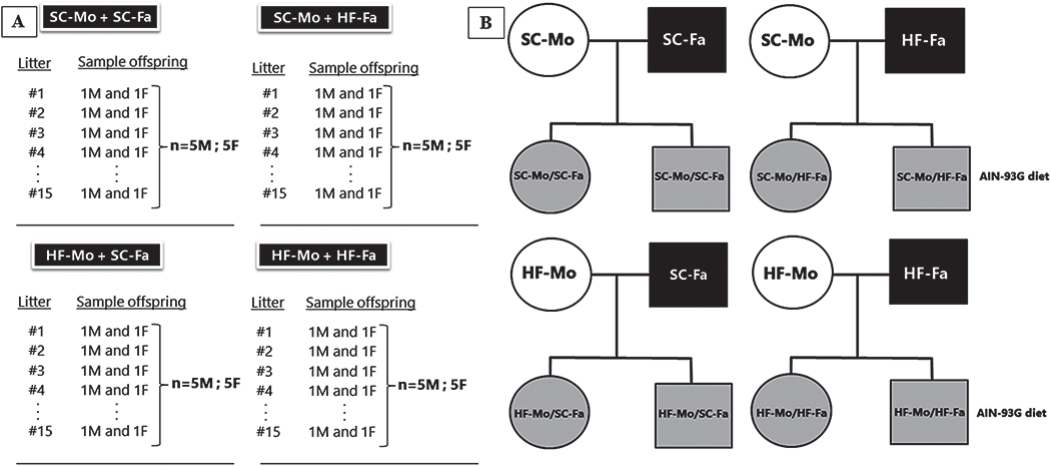
Sampling design and formation of the groups. (A) Sampling design for the study and (B) Scheme of the experimental groups. The letters M and F represent the male and female offspring, respectively (in A). Circles represent the female sex, and squares represent the male sex (in B). Abbreviations: Standard-chow group (SC); high-fat group (HF); father (Fa); mother (Mo). Groups—Father that received standard chow (SC-Fa); Father that received high-fat diet (HF-Fa); mother that received standard chow (SC-Mo); mother that received high-fat diet (HF-Mo); offspring of SC mother and SC father (SC-Mo/SC-Fa); offspring of SC mother and HF father (SC-Mo/HF-Fa); offspring of HF mother and SC father (HF-Mo/SC-Fa); offspring of HF mother and HF father (HF-Mo/HF-Fa).

At 12-weeks old, one male and one female from each group were crossed for mating. Thus, we obtained four groups of progenitors—Mother (Mo) and Father (Fa) that were exposed to different diet combinations: SC-Mo/SC-Fa (15 mothers and 15 fathers), SC-Mo/HF-Fa (15 mothers and 15 fathers), HF-Mo/SC-Fa (15 mothers and 15 fathers), and HF-Mo/HF-Fa (15 mothers and 15 fathers), as shown in Fig 1. Males were withdrawn immediately after the appearance of a vaginal plug, which denoted day one of pregnancy.

The routine of experimental diets was continued during the pregnancy and lactation periods. Mother and father body mass (BM) were measured weekly, and BM gain was calculated during the pre-mating period (after eight weeks of the experimental diet). Fresh chow was provided daily, and food intake was evaluated during the entire experimental period. Food intake was determined as the difference between the food supplied, and the amount of food left in the cage and energy intake was determined as the product of food consumption by the energy content of the diet.

Litters were standardized to six pups immediately after birth (three males and three females, when possible) and stayed with the respective mother until weaning. The fathers and mothers were sacrificed after weaning. One male and one female offspring of each litter were removed to form the experimental groups. The offspring were identified according to sex, with maternal diet (first letters) and paternal diet (second letters) and received a standard chow diet (SC/AIN-93G) until the age of 12-weeks, when they were sacrificed (Fig 1 illustrates the experimental design and the different groups).

From the birth to 12-weeks old, BM of offspring was measured weekly, and weight gain was calculated during this period. The food intake and energy intake of offspring were also measured.

### Oral glucose tolerance test (OGTT)

An OGTT was performed in HF and SC fathers and HF and SC mothers two days before mating and in the offspring at 12-weeks old (three days prior to the euthanasia), after 6-h of food deprivation (1a. m.– 7a. m.). Glucose (25% in sterile saline, 0.9% NaCl) at a dose of 1 g/kg was administered by orogastric gavage to provoke glucose overload, and caudal vein blood samples were collected at 0, 15, 30, 60 and 120 min after glucose administration. Blood glucose was measured using a glucometer (Accu-Chek, Roche, Germany). Glucose values at time zero were considered fasting glucose levels. In this analysis, the area under the curve (AUC) was calculated from zero to 120 min using the trapezoid rule and was measured in arbitrary units (a. u.) to assess glucose tolerance (GraphPad Prism v. 6.05 for Windows; GraphPad Software, La Jolla, CA, USA).

### Euthanasia

Fathers and mothers were sacrificed after offspring weaning. In turn, the offspring was sacrificed at 12-weeks old. The animals were fasted overnight (food-deprived from 1a. m.– 7a. m.) and then deeply anesthetized (sodium pentobarbital, 150 mg/kg intraperitoneal). Blood samples were rapidly obtained by cardiac puncture, and centrifugation separated the plasma (120 g for 15 min) that was stored at -20°C until analyzes could be performed.

We dissected and weighed the inguinal and intra-abdominal fat pads. The inguinal fat pad as kept as the subcutaneous fat located between the lower part of the rib cage and the mid-thigh. The intra-abdominal fat pad was held as the retroperitoneal fat attached to the posterior abdominal wall near the kidneys plus the genital fat located in the lower part of the abdomen that is connected to the epididymis in males and connected to the ovaries and oviducts in females. The adiposity index was determined as the ratio between the sum of the fat masses divided by the total BM (as a percentage) [16].

Liver of offspring was removed, weighed, and fragments of all lobes were put in fixative (formaldehyde 4% w/v, 0.1 M phosphate buffer pH 7. 2) for light microscopy. Other fragments were frozen at −80°C for further analyzes.

### Liver histology

Liver fragments of offspring were embedded in Paraplast plus (Sigma-Aldrich Co., St. Louis, MO, USA), sectioned at a nominal thickness of five micrometers, and stained with hematoxylin and eosin. Digital images were obtained with a Leica DMRBE microscope (Leica Microsystems GmbH, Wetzlar, Germany) and an Infinity 1-5c camera (Lumenera Co., Ottawa, ON, Canada). The volume densities (Vv) of steatosis were estimated by point counting as described elsewhere [17,18]. Briefly, Vv[steatosis] was estimated with a test-system made up of 36 test points produced by the STEPanizer web-based system [19]. The following equation was employed: Vv_[steatosis; liver]_: Pp_[steatosis; liver]_/P_T_ (Pp is the number of points that hit the structure, and P_T_ is the total test points). In addition, the frozen fragments of the liver were used for measuring liver triacylglycerol. Briefly, 50 mg of frozen liver tissue was placed in an ultrasonic processor with 1 ml of isopropanol. The homogenate was centrifuged at 2000 g, and 5 μl of the supernatant was used to measure liver triacylglycerol with a kit and an automated spectrophotometer (Bioclin System II, Quibasa Ltda., Belo Horizonte, Brazil) [18].

### Plasma analysis

Total cholesterol and triacylglycerol of fathers, mothers and offspring were measured with an automatic spectrophotometer using a commercial kit (Bioclin System II, Quibasa, Belo Horizonte, MG, Brazil). Insulin and adiponectin concentrations of fathers, mothers and offspring were analyzed in duplicate using commercially available enzyme-linked immunosorbent assay kits (Rat/Mouse Insulin ELISA kit Cat. #EZRMI-13K, Rat/Mouse Adiponectin ELISA kit Cat. #EZMADP-60K, Millipore, Missouri, USA), using Fluostar Omega equipment (BMG Labtech GmbH, Germany).

### RT-qPCR

Total RNA was extracted from approximately 50 mg of liver tissue using Trizol reagent (Invitrogen, CA, USA). The RNA amount was determined using Nanovue (GE Life Sciences) spectroscopy, and 1 mg of RNA was treated with DNAse I (Invitrogen). Synthesis of the first strand cDNA was performed using Oligo (dT) primers for mRNA and Superscript III reverse-transcriptase (both Invitrogen). Quantitative real-time PCR (RT-qPCR) was performed using a Biorad CFX96 cycler and the SYBR Green mix (Invitrogen). Primers for RT-qPCR (carnitine palmitoyltransferase I—CPT-1, peroxisome proliferator activator receptor alpha-PPAR-alpha, sterol regulatory element binding protein 1-c—SREBP-1c, fatty acid synthase—FAS, phospho-enol-pyruvate-carboxykinase—PEPCK,glucose-6-phosphatase—G6Pase, and beta-actin- β-actin) were designed using the Primer3 online software and are indicated in Table 2. Endogenous control beta-actin was used to normalize the expression of the selected genes. Efficiencies of RT-qPCR for the target gene and the endogenous control were approximately equal and were calculated through dilution series of cDNA. Real-time PCR reactions were conducted as follows: after a pre-denaturation and polymerase-activation program (4 min at 95°C), forty-four cycles each consisting of 95°C for 10 s and 60°C for 15 s were followed by a melting curve program (60 to 95°C with heating rate of 0.1°C/s). Negative controls consisted of wells in which cDNA was substituted for deionized water. The relative expression ratio (RQ) of mRNA was calculated by the equation 2^-ΔΔCt^, in which -ΔCT expresses the difference between the number of cycles (CT) of the target genes and the endogenous control.

**Table 2:** RT-qPCR primers and respective sequences.

Name	5–3’	Primers
CPT-1	FW	AAGGAATGCAGGTCCACATC
CPT-1	RV	CCAGGCTACAGTGGGACATT
PPAR-α	FW	TCGAGGAAGGCACTACACCT
PPAR-α	RV	TCTTCCCAAAGCTCCTTCAA
SREBP-1c	FW	TCTGCCTTGATGAAGTGTGG
SREBP-1c	RV	AGCAGCCCCTAGAACAAACA
FAS	FW	CATCCAGATAGGCCTCATAGAC
FAS	RV	CTCCATGAAGTAGGAGTGGAAG
PEPCK	FW	AGCTGCATAATGGTCTGG
PEPCK	RV	GAACCTGGCGTTGAATGC
G6Pase	FW	AACGCCTTCTATGTCCTCTTTC
G6Pase	RV	GTTGCTGTAGTAGTCGGTGTCC
β-actin	FW	TGAGACCTTCAACACCCCAGCCA
β-actin	RV	CGTAGATGGGCACAGTGTGGGTG

Abbreviations: carnitine palmitoyltransferase I (CPT-1); peroxisome proliferator activator receptor (PPAR)-alpha, sterol regulatory element binding protein (SREBP)-1c; fatty acid synthase (FAS); phosphoenolpyruvate carboxykinase (PEPCK); glucose-6-phosphatase (G6Pase).

### Liver immunoblotting

The total hepatic proteins of offspring were extracted in homogenizing buffer and protease inhibitors. Next, the homogenates were centrifuged for 20 min at 4°C and the supernatants were collected. Equal quantities of total protein were re-suspended in sodium dodecyl sulfate (SDS) containing sample buffer, heated for 5 min at 100°C, and separated by SDS-polyacrylamide gel electrophoresis. After electrophoresis, proteins were electroblotted onto a polyvinyl difluoride transfer membrane (Amersham Biosciences, Piscataway, NJ). In addition, the efficiency of the transfer was visualized by Ponceau solution staining. The membrane was blocked by incubation with nonfat dry milk (6% in Tween 20–Tris-buffered saline). Antibodies against sterol regulatory element binding protein-1c (SREBP-1c, 68 kDa), peroxisome proliferator-activated receptor alpha (PPAR-alpha, 55 kDa), fatty acid synthase (FAS, 270 kDa) and beta-actin (beta-actin, 43 kDa) were purchased from Santa Cruz (Santa Cruz Biotechnology, CA, USA). Following incubation with the primary antibody, the membrane was incubated with the secondary antibody for 1 h at room temperature. The membrane was developed using ECL Western blotting detection reagents, and the images of the blots were obtained with the Bio-Rad Molecular Imaging ChemiDoc XRS System (Bio-Rad, Hercules, CA, USA). The intensity of the chemiluminescent bands was quantified using ImageJ software, version 1.49i (NIH, imagej.nih.gov/ij, USA). The blots were stripped and reprobed for beta-actin as a loading control to normalize the blot data.

### Statistical analysis

Data are expressed as the mean and standard deviation and were tested for normality and homoscedasticity of the variances. The differences among groups were analyzed using one-way ANOVA followed by the posthoc test of Holm-Sidak (to test data between SC- and HF diet-fed mothers and fathers; and to study male and female offspring) (GraphPad Prism v. 6.05 for Windows, GraphPad Software, La Jolla, CA, USA). Interactions between paternal and maternal diets and offspring sex were evaluated through three-way ANOVA (2 x 2 x 2 factorial) (Statistica, v. 7; StatSoft Inc., Tulsa, OK, USA). The overall significance of a three-way effect and critical two-way interactions (paternal diet- offspring sex, maternal diet- offspring sex and paternal diet-maternal diet) were examined. In all cases, *P*<0.05 was considered statistically significant.

## Results

### Father and Mother Data

All females used in this study became pregnant, and they all birthed live litters.

#### Body mass and food consumption

There were no significant differences in the initial BM of fathers and mothers of the SC and HF groups. However, after eight weeks (the pre-mating period), the HF-father was heavier than the SC-father (+24%; *P*<0.0001); the HF-mother was heavier than the SC-mother (+21%; *P* = 0.0005) (Table 3).

**Table 3:** Data of fathers and mothers fed standard chow (SC) and high-fat (HF) diet.

Data	Fathers		Mothers	
	SC	HF	SC	HF
Initial body mass (g)	16.20±1. 30^*a*^	16.50±1. 13^*a*^	10.90±0.98^*a*^	11.03±0.83^*a*^
Pre-mating body mass (g)	29.07±0.81^*a*^	36.04±0.62^*b*^	23.38±0.61^*c*^	28.20±0.45^*d*^
Food intake (g/day/mouse)	3.47±0.25^*a*^	3.42±0.05^*a*^	2.09±0.03^*a*^	2.07±0.12^*a*^
Energy intake (kJ/day/mouse)	57.29±4. 08^*a*^	70.76±1. 02^*b*^	34.51±0.59^*c*^	42.83±2. 60^*d*^
Fasting glucose (mmol/L)	4.77±0.76^*a*^	6.62±0.45^*b*^	5.48±0.41^c^	7.26±0.78^*d*^
Total cholesterol (mg/dL)	114.30±5. 75^*a*^	144.80±16. 12^*b*^	92.01±8. 94^*c*^	117.20±2. 82^*d*^
Triglycerides (mg/dL)	80.18±1.37^*a*^	96.15±4.45^*b*^	72.58±6.02^*c*^	83.13±7.17^*d*^
Insulin (pg/mL)	301.00±27. 42^*a*^	531.80±21.76^*b*^	230.30±6.90^*c*^	338.80±26.21^*d*^
Adiponectin (10^6^ pg/mL)	8.34±0.74^*a*^	4.65±1. 49^*b*^	10.73±0.21^*c*^	6.49±0.69^*d*^

Data are expressed as the mean and SD (n = 5 mice per group, one way ANOVA and the post hoc test of Holm-Sidak). Same letters represent equal groups, with no statistical difference while different letters represent different groups from each other, with statistical difference (*P*<0.05). Abbreviations: father that received standard chow (SC-Fa); father that received high-fat diet (HF-Fa); mother that received standard chow (SC-Mo); mother that received high-fat diet (HF-Mo).

**Fig 2:**
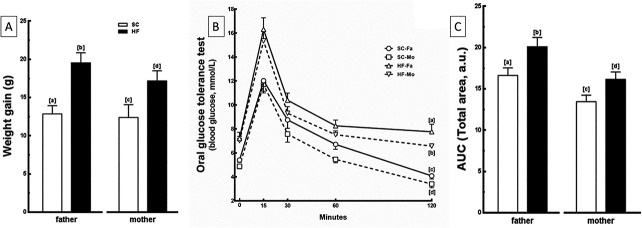
Parental data. (A) Pre-mating weight gain, (B) Oral glucose tolerance test (OGTT) and (C) Area under the curve (AUC) from OGTT of fathers and mothers. Data are expressed as the mean and SD for n = 5 mice per group in all analyzes (one way ANOVA and the posthoc test of Holm-Sidak). Same letters represent equal groups with no statistical difference while different letters represent different groups from each other, with statistical difference (P<0.05). Abbreviations: father that received standard chow (SC-Fa); father that received high-fat diet (HF-Fa); mother that received standard chow (SC-Mo); mother that received high-fat diet (HF-Mo); area under the curve (AUC) and arbitrary units (a. u.).

In relation to BM evolution during the pre-mating period, the HF-father had a BM 52% higher and the HF-mother had a BM 39% higher compared with their control counterparts (*P*<0.0001) (Fig 2).

The food intake measured during the experimental period was not different among the groups, however, energy intake during the same period was 24% greater in the HF-father and the HF-mother than in the SC-father and SC-mother, respectively (*P*<0.0001) (Table 3).

#### Fasting glucose and OGTT

In the pre-mating period, HF-father and HF-mother groups presented hyperglycemia (+39% and +33%, respectively; *P* = 0.0006) (Table 3) and glucose intolerance, demonstrated by the highest peak in glucose levels after 15 min of oral glucose administration and the greatest delay in the time-course of glucose clearance (*P*<0.0001). Finally, this condition was corroborated by the higher values of the area under the curve in these groups compared to the SC groups (+20% in both; *P* = 0.006) (Fig 2).

#### Plasma analysis

HF-father and HF-mother groups presented hypercholesterolemia (+27% in both; *P* = 0.005) and hypertriglyceridemia (+20% and +15%, respectively; *P* = 0.04). Insulin values were higher in the HF-father (+77%; *P*<0.0001) and in the HF-mother (+47%; *P*<0.0001) than in the SC-father and the SC-mother groups, which is consistent with glucose intolerant OGTT data. In addition, adiponectin values were 44% lower in HF-father and 40% lower in HF-mother than in their counterparts (*P* = 0.0001). These data are summarized in Table 3.

### Offspring data

#### Birth outcomes

The litter size was not affected by the diets of the father and/or of the mother. The number of pups per litter, median (confidence interval 95%) was: a) SC-Mo/SC-Fa, 8 (0.18), b) SC-Mo/HF-Fa, 8 (0.18), c) HF-Mo/SC-Fa, 8 (0.17), d) HF-Mo/HF-Fa, 7 (0.15). In a few litters, some newborns were lost, but the formation of the groups was not affected.

#### Body mass and food consumption

At birth, the offspring who had both a HF-mother and HF-father were the heaviest in comparison with the other groups. HF-Mo/HF-Fa offspring were heavier than SC-Mo/HF-Fa (+22% in male and +16% in female; *P*<0.0001) and heavier than HF-Mo/SC-Fa offspring (+19% in male and +13% in female; *P* = 0.007) (Table 4). However, there was no effect of maternal or paternal obesity on birth weight of the offspring.

**Table 4:** Characteristics of male and female offspring of fathers and mothers that received standard chow (SC) and/or high-fat (HF) diet.

Male	SC-Mo/SC-Fa	SC-Mo/HF-Fa	HF-Mo/SC-Fa	HF-Mo/HF-Fa
Initial body mass (g)	1.20±0.04^*a*^	1.23±0.08^*a*^	1.26±0.05^*a*^	1.50±0.05 ^*b*^
Body mass at weaning (g)	9.80±0.85^*a*^	9.90±0.58^*a*^	11.50±0.67^*b*^	12.68±0.77^*c*^
Final body mass (g)	25.03±0.35^*a*^	25.53±0.97^*a*^	28.60±1.10^*b*^	31.72±0.88^*c*^
Food intake (g/day/mouse)	2.20±0.32^*a*^	2.36±0.38^*a*^	3.49±0.43^*b*^	4.40±0.34^*c*^
Energy intake (kJ/day/mouse)	36.32±5.30^*a*^	38.97±6.36^*a*^	57.62±4.82^*b*^	72.65±5.53^*c*^
Fasting glucose (mmol/L)	4.55±0.52^*a*^	6.69±0.35^*b*^	8.12±0.48^*b*^	10.21±1.07^*c*^
Adiposity Index (%)	2.42±0.27^*a*^	2.40±0.27^*a*^	3.86±0.53^*b*^	5.39±0.45^*c*^
Total cholesterol (mg/dL)	67.67±3.14^*a*^	70.45±13.10^*a*^	90.20±2.65^*b*^	112.90±6.36^*c*^
Triacylglycerol (mg/dL)	54.75±1.08^*a*^	57.00±7.50^*a*^	65.77±4.39^*b*^	79.90±6.05^*c*^
Insulin (pg/mL)	88.60±3.75^*a*^	140.20±9.29^*b*^	243.50±33.27^*c*^	377.80±7.62^*d*^
Adiponectin (10^6^ pg/mL)	7.40±0.43^*a*^	6.04±0.50^*b*^	4.65±0.88^*c*^	3.22±0.29^*d*^
Hepatic mass (g/cm)	0.34±0.04^*a*^	0.53±0.04^*b*^	0.50±0.06^*b*^	0.70±0.05^*c*^
Liver TG (mg/dl/mg)	2.79±0.46^*a*^	4.71±0.89^*b*^	4.90±0.77^*b*^	6.60±0.34^*c*^
**Female**				
Initial body mass (g)	1.13±0.03e	1.14±0.04e	1.20±0.10e	1.36±0.05f, *
Body mass at weaning (g)	8.96±0.85e,*	8.99±0.58e,*	10.01±0.67f,*	11.90±0.78g,*
Final body mass (g)	19.13±0.39e,*	19.18±0.63e,*	21.37±0.83f,*	23.47±0.74g,*
Food intake (g/day/mouse)	1.98±0.50e	1.98±0.42e	2.76±0.38f,*	3.52±0.77g,*
Energy intake (kJ/day/mouse)	32.69±8.20e	32.69±6.94e	45.57±6.26f,*	58.12±12.76g,*
Fasting glucose (mmol/L)	3.97±0.35e	5.72±0.26f	6.04±1.04f,*	7.78±0.81g,*
Adiposity Index (%)	3.14±0.06e	3.00±0.17e	4.85±0.20f,*	6.93±0.30g,*
Total cholesterol (mg/dL)	58.17±2.48e	60.13±6.46e	76.83±4.75f, *	95.62±8.43g,*
Triacylglycerol (mg/dL)	53.60±2.67e	55.46±1.43e	60.65±1.90e,*	71.13±2.55f,*
Insulin (pg/mL)	85.98±6.27e	136.60±36.49f	198.40±21.82g,*	279.80±10.90h,*
Adiponectin (106 pg/mL)	8.30±0.51e	7.02±0.48f	5.66±0.90g,*	4.29±0.43h,*
Hepatic mass (g/cm)	0.32±0.04e	0.48±0.05f	0.42±0.07f	0.61±0.07g
Liver TG (mg/dl/mg)	2.64±0.13e	4.33±0.76f	4.46±0.80f	6.25±1.14g

All offspring groups received postnatal SC diet (n = 5). Data are expressed as the mean and SD (n = 5 mice per group, one way ANOVA and the post hoc test of Holm-Sidak). Same letters represent equal groups, with no statistical difference while different letters represent different groups from each other, with statistical difference (*P*<0.05)

* different from the corresponding counterpart (*P*<0.05).

Abbreviations: offspring of SC mother and SC father (SC-Mo/SC-Fa); offspring of SC mother and HF father (SC-Mo/HF-Fa); offspring of HF mother and SC father (HF-Mo/SC-Fa); offspring of HF mother and HF father (HF-Mo/HF-Fa); triacylglycerol (TG).

**Fig 3:**
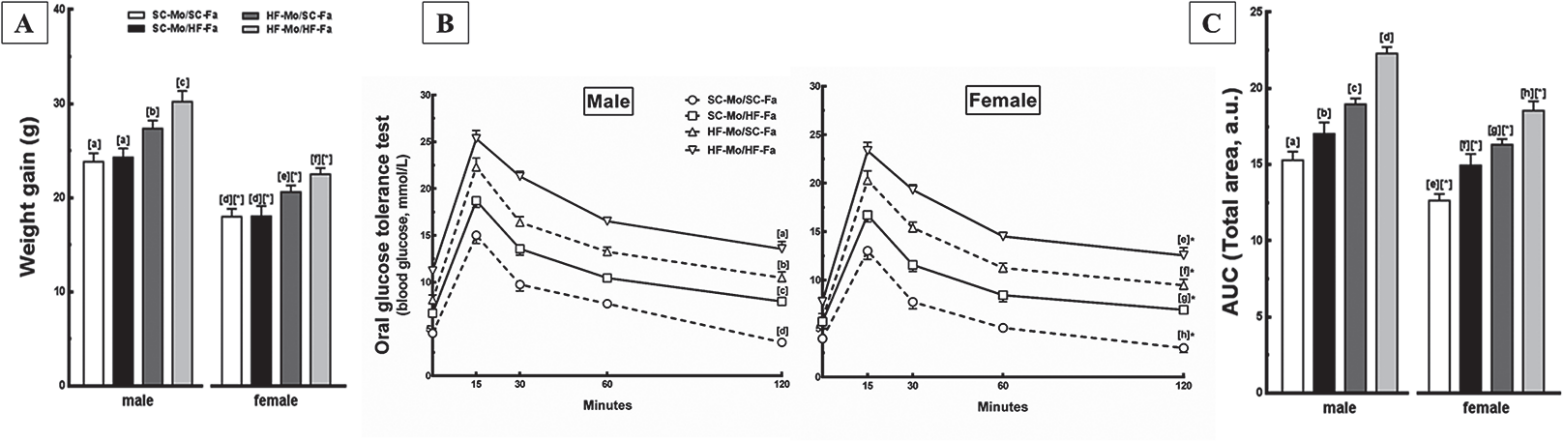
Offspring data. (A) Total weight gain, (B) Oral glucose tolerance test (OGTT) and (C) Area under the curve (AUC) of OGTT of the male and female offspring at 12-weeks old. Data are expressed as the mean and SD for N = 5 mice per group in all analyzes (one way ANOVA and the posthoc test of Holm-Sidak). Same letters represent equal groups with no statistical difference while different letters represent different groups from each other, with statistical difference (*P*<0.05), * different from the corresponding counterpart (*P*<0.05). Abbreviations: father that received standard chow (SC-Fa); father that received high-fat diet (HF-Fa); mother that received standard chow (SC-Mo); mother that received high-fat diet (HF-Mo). Offspring of SC mother and SC father (SC-Mo/SC-Fa); offspring of SC mother and HF father (SC-Mo/HF-Fa); offspring of HF mother and SC father (HF-Mo/SC-Fa); offspring of HF mother and HF father (HF-Mo/HF-Fa); area under the curve (AUC) and arbitrary units (a. u.).

At weaning, the male and female HF-Mo/HF-Fa offspring continued to be the heaviest, and this condition persisted throughout the experiment (*P*<0.001). In addition, these offspring presented maximized values of BM gain, with males being more affected than females (*P*<0.001; three-way ANOVA, Fig 3).

The HF-Mo/SC-Fa offspring presented an increase in BM at weaning, continuing until the end of the experiment (*P*<0.0001). These results are corroborated by higher BM gain, especially in males, arising from the interaction between maternal diet and offspring sex (*P*<0.05; two-way ANOVA, Fig 3). Meanwhile, the HF-father not influenced this parameter (Fig 3 and Table 4).

In our results, the HF-Mo/HF-Fa and HF-Mo/SC-Fa offspring showed increased food intake and, consequently, higher energy intake. The HF-father did not affect this variable (Table 4). When both the mother and the father received a HF diet, the HF-Mo/HF-Fa offspring showed markedly higher food intake in relation to SC-Mo/HF-Fa (+86% in male and +78% in female; *P*<0.0001) and HF-Mo/SC-Fa (+26% in male and +28% in female; *P* = 0.007) (Table 4). These findings are supported by a three-way ANOVA test that showed an interaction between maternal diet, paternal diet and offspring sex, with males being more affected than the female offspring (*P*<0.0001; three-way ANOVA).

The HF-Mo/SC-Fa offspring had hyperphagia in comparison with the SC-Mo/SC-Fa offspring (+59% in male and +39% in female; *P* = 0.0001) and SC-Mo/HF-Fa offspring (+48% in male and +39% in female; *P* = 0.0001) (Table 4). The two-way ANOVA test showed that maternal diet and offspring sex affected these variables, with males being more influenced than females (*P*<0.005; two-way ANOVA).

#### Fasting glucose and Oral Glucose Tolerance Test

Again, the male and female HF-Mo/HF-Fa offspring had maximized values of fasting glucose and glucose intolerance, with the highest peak in glucose levels after 15 min of oral glucose administration and the most notorious delay in time-course of glucose clearance, remaining elevated for 120 min after glucose administration (*P*<0.0001, Fig 3 and Table 4), being males more affected than females (*P*<0.0001; three-way ANOVA).

Interestingly, the HF-father also changed these parameters in an independent way. When only the father received a HF diet, the SC-Mo/HF-Fa offspring had hyperglycemia (+46% in both sexes; *P* = 0.006) (Table 4) and glucose intolerance, demonstrated by a higher peak in glucose levels and greater delay in the time-course of glucose clearance (*P*<0.0001) in comparison to SC-Mo/SC-Fa. Furthermore, male and female offspring of the HF-father had an increase of 25% in the area under the curve for OGTT in both sexes compared to SC-Mo/SC-Fa offspring (*P* = 0.0007) (Fig 3).

The HF-Mo/SC-Fa offspring also presented this deleterious phenotype, being more affected than the offspring of HF-father (*P*<0.001) (Fig 3 and Table 4). The two-way ANOVA test showed that the paternal and maternal HF diet and offspring sex affected these results because males were more affected by glucose intolerance (*P*<0.05; two-way ANOVA).

#### Adiposity Index

Offspring from both HF-mothers and HF-fathers and offspring of HF-mothers had a higher adiposity index while HF-father did not influence this variable. The HF-Mo/HF-Fa offspring had higher adiposity index values in relation to SC-Mo/HF-Fa offspring (+125% in male and +131% in female; *P*<0.0001) and in relation to HF-Mo/SC-Fa offspring (approximately +40%, both sexes; *P*<0.0001) (Table 4), being the females more sensitive to diet-induced obese parents (*P*<0.001; three-way ANOVA).

The adiposity index was higher in HF-Mo/SC-Fa offspring than SC-Mo/SC-Fa and SC-Mo/HF-Fa offspring (*P* = 0.002) (Table 4), especially in females offspring (*P*<0.0001; two-way ANOVA).

#### Liver

The HF-Mo/HF-Fa offspring presented the highest liver mass, highest content of triacylglycerol and steatosis, implying an interaction between paternal diet and maternal diet (*P*<0.0001; two-way ANOVA), being males and females likewise influenced by diet-induced obese parents.

**Fig 4:**
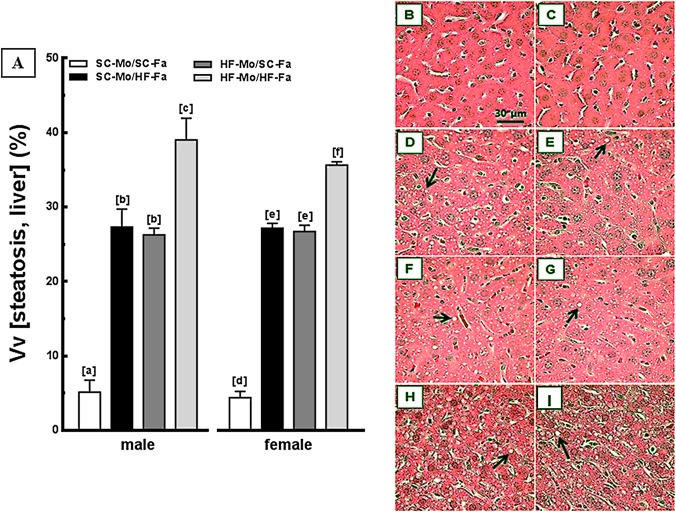
Offspring data: liver. (A) Volume densities (Vv) of steatosis (left frame, n = 5 mice per group) and photomicrographs of the liver (right frame, B-I) from male and female offspring at 12-weeks old (HE staining). Data are expressed as the mean and SD (one-way ANOVA and the posthoc test of Holm-Sidak). Same letters represent equal groups with no statistical difference while different letters represent different groups from each other, with statistical difference (*P*<0.05). Photomicrographs: B and C—male and female offspring of SC mother and SC father, respectively (SC-Mo/SC-Fa); D and E—male and female offspring of SC mother and HF father, respectively (SC-Mo/HF-Fa); F and G—male and female offspring of HF mother and SC father, respectively (HF-Mo/SC-Fa); H and I—male and female offspring of HF mother and HF father, respectively (HF-Mo/HF-Fa). Offspring of obese mother or obese father and both obese parents present numerous hepatocytes with fat droplets (arrows).

The HF-father and HF-mother equally affected the liver phenotype in male and female offspring. The SC-Mo/HF-Fa offspring showed increased hepatic mass (approximately +50% in both sexes; *P*<0.0001, Table 4), triacylglycerol content (approximately +64%, both sexes; *P*<0.0001, Table 4) and significant steatosis (+540%, both sexes; *P*<0.0001, Fig 4) in relation to SC-Mo/SC-Fa offspring. A similar deleterious pattern was observed in the HF-Mo/SC-Fa offspring, without sex differences (Fig 4 and Table 4).

#### Plasma analysis

The offspring of both HF-mother and HF-father and offspring of HF-mother had higher total cholesterol and triacylglycerol plasma levels while HF-father did not influence this lipid profile. Total cholesterol was approximately 60% higher in HF-Mo/HF-Fa than in SC-Mo/HF-Fa offspring (*P*<0.0001 in both sexes) and 25% greater than in HF-Mo/SC-Fa offspring (*P*<0.003 in both sexes) (Table 4). HF-Mo/HF-Fa offspring showed higher values of triacylglycerol in relation to SC-Mo/HF-Fa (+40% in male and +28% in female; *P*<0.0001) and HF-Mo/SC-Fa offspring (+22% in male and +17% in female; *P* = 0.002) (Table 4), with males being more affected than female offspring (*P*<0.0001; three-way ANOVA).

Total cholesterol was higher in HF-Mo/SC-Fa offspring than in SC-Mo/SC-Fa and in SC-Mo/HF-Fa groups (*P*<0.05) (Table 4). Interestingly, triacylglycerol levels were higher only in male offspring in comparison with other groups (*P* = 0.02) (Table 4). The two-way ANOVA test showed that the lipid profile was affected by maternal diet and offspring sex, with greater impairment in males (*P*<0.0001; two-way ANOVA).

As occurred with fasting glucose, the HF-mother and/or HF-father influenced the insulin levels of the offspring. The HF-Mo/HF-Fa offspring showed higher values of insulin in relation to SC-Mo/HF-Fa offspring (+170% in male and +105% in female; *P*<0.0001) and in HF-Mo/SC-Fa offspring (+55% in male and +41% in female; *P* = 0.0002) (Table 4), mainly in males offspring (*P*<0.001; three-way ANOVA).

The SC-Mo/HF-Fa offspring showed hyperinsulinemia (+58% in both sexes; *P* = 0.04) compared to SC-Mo/SC-Fa (Table 4), with male offspring being more influenced by the HF-father than females (*P*<0.0001; two-way ANOVA). In addition, the HF-mother also affected the insulin level of offspring, being greater than in SC-Mo/HF-Fa offspring (+74% in male and +45% in females; *P* = 0.006) (Table 4). The maternal diet and offspring sex altered this parameter, especially in the males (*P*<0.0001; two-way ANOVA).

Both HF-mother and/or HF-father influenced the plasma concentration of adiponectin in the offspring. The HF-Mo/HF-Fa offspring had lower values of adiponectin in relation to SC-Mo/HF-Fa (-47% in male and -39% in female; *P*<0.0001) and in relation to HF-Mo/SC-Fa (approximately -31% in both sexes; *P*<0.04, Table 4). This pattern was more evident in males than in females offspring (*P*<0.01; three-way ANOVA).

The adiponectin level was approximately 15% lower in SC-Mo/HF-Fa offspring than in SC-Mo/SC-Fa (*P*<0.05 in both sexes). Nevertheless, this decrease was less pronounced than in HF-Mo/SC-Fa offspring (Table 4). The parental diet and the offspring sex affected adiponectin level. Paternal and maternal diets interacted to affect the adiponectin level, with males being more affected than females (*P*<0.05; two-way ANOVA).

#### RT-qPCR

**Fig 5:**
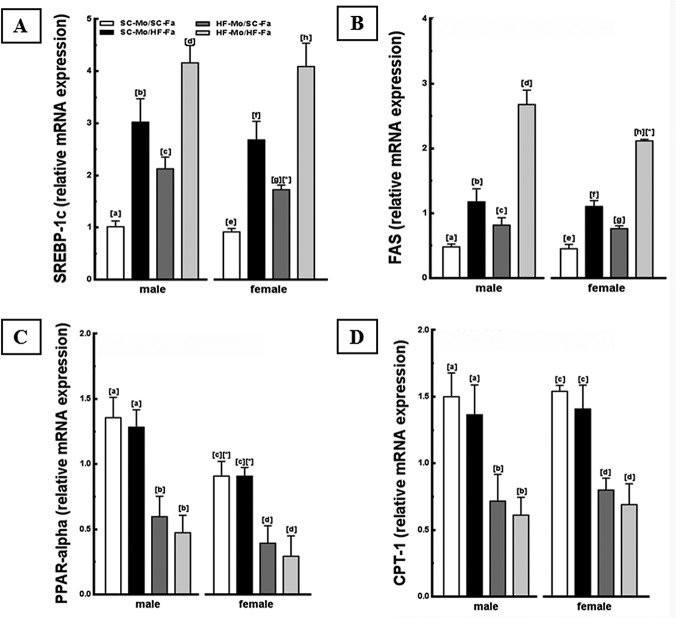
Molecular analyses in the offspring liver (panel 1). (A) SREBP-1c, (B) FAS, (C) PPAR-alpha and (D) CPT-1 mRNA levels of the male and female offspring at 12-weeks old. Endogenous control beta-actin was used to normalize the expression of the selected genes. Data are expressed as the mean and SD (N = 5 mice per group, one-way ANOVA and the posthoc test of Holm–Sidak). Same letters represent equal groups with no statistical difference while different letters represent different groups from each other, with statistical difference (*P*<0.05), * different from the corresponding counterpart (*P*<0.05). Abbreviations: sterol regulating element binding protein (SREBP-1c); fatty acid synthase (FAS); peroxisome proliferator activator receptor alpha (PPAR-alpha); carnitine palmitoyltransferase I (CPT-1); offspring of SC mother and SC father (SC-Mo/SC-Fa); offspring of SC mother and HF father (SC-Mo/HF-Fa); offspring of HF mother and SC father (HF-Mo/SC-Fa); offspring of HF mother and HF father (HF-Mo/HF-Fa).

The HF-mother and/or HF-father led to differential expression of some genes related to lipogenesis (SREBP-1c and FAS) and hepatic beta-oxidation (PPAR-alpha, CPT-1) in both sexes offspring. The male and female HF-Mo/HF-Fa offspring showed higher expressions of these lipogenic genes in relation to other groups, findings supported by the interaction between paternal diet and maternal diet (*P*<0.001; two-way ANOVA, Fig 5). Inversely, lower expressions of PPAR-alpha and CPT-1 were observed in the male and female HF-Mo/HF-Fa offspring (*P*<0.001, Fig 5), similarly to what happened to the offspring of HF-mother, indicating that parental programming has an influence on hepatic beta-oxidation.

In addition, the SC-Mo/HF-Fa offspring had higher SREBP-1c and FAS expression compared to SC-Mo/SC-Fa offspring (*P*<0.0001, Fig 5). Interestingly, SC-Mo/HF-Fa offspring had higher expression of SREBP-1c (+42% in male and +55% in female; *P* = 0.01, Fig 5) and FAS (+45%, both sexes; *P* = 0.01, Fig 5) in relation to HF-Mo/SC-Fa offspring. The two-way ANOVA test showed that father diet and offspring sex collaborated with the elevation in SREBP-1c, with male offspring being more influenced than their female counterparts (*P*<0.001; two-way ANOVA). The HF-father did not alter the gene expression of PPAR-alpha and CPT-1 in male and female offspring.

**Fig 6:**
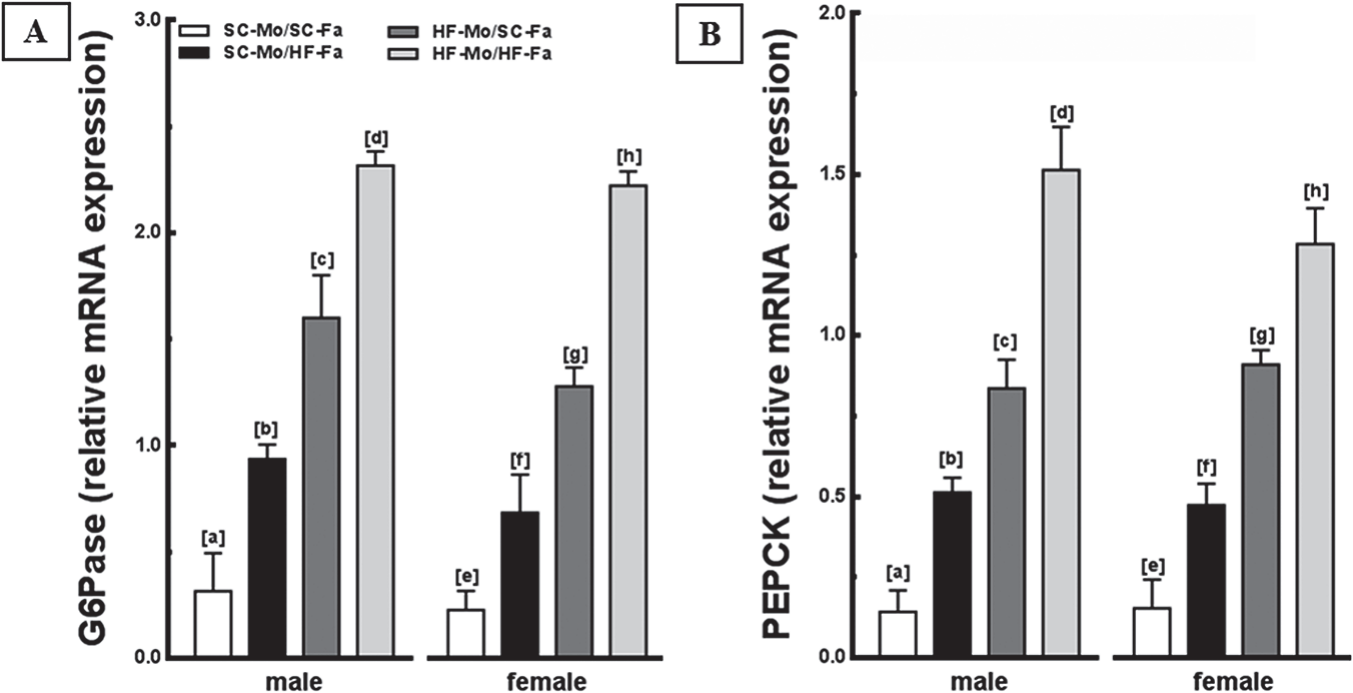
Molecular analyses in the offspring liver (panel 2). (A) G6Pase and (B) PEPCK mRNA levels of the male and female offspring at 12-weeks old. Endogenous control beta-actin was used to normalize the expression of the selected genes. Data are expressed as the mean and SD (n = 5 mice per group, one-way ANOVA and the posthoc test of Holm–Sidak). Same letters represent equal groups with no statistical difference while different letters represent different groups from each other, with statistical difference (*P*<0.05), * different from the corresponding counterpart (*P*<0.05). Abbreviations: phosphoenolpyruvate carboxykinase (PEPCK); glucose-6-phosphatase (G6Pase); offspring of SC mother and SC father (SC-Mo/SC-Fa); offspring of SC mother and HF father (SC-Mo/HF-Fa); offspring of HF mother and SC father (HF-Mo/SC-Fa); offspring of HF mother and HF father (HF-Mo/HF-Fa).

The expression of G6Pase and PEPCK enzymes were assessed to verify the hepatic glucose production and insulin resistance. As a result, both HF-mother and/or HF-father influenced the expression of these enzymes in the offspring. In both sexes HF-Mo/HF-Fa offspring, the genes were overexpressed, which supports the interaction between paternal and maternal diets on hepatic glucose production and insulin resistance (*P*<0.0001; two-way ANOVA, Fig 6). Additionally, the SC-Mo/HF-Fa offspring had higher expression of G6Pase (+200%, both sexes; *P*<0.0001, Fig 6), and PEPCK (+261% in male and +208% in females; *P*<0.0001, Fig 6), compared to SC-Mo/SC-Fa offspring. Finally, both male and female HF-Mo/SC-Fa offspring also overexpressed both G6Pase and PEPCK, being even more affected than the offspring of HF-father (*P*<0.0001, Fig 6). There were no sex differences in these variables.

#### Western Blotting

**Fig 7:**
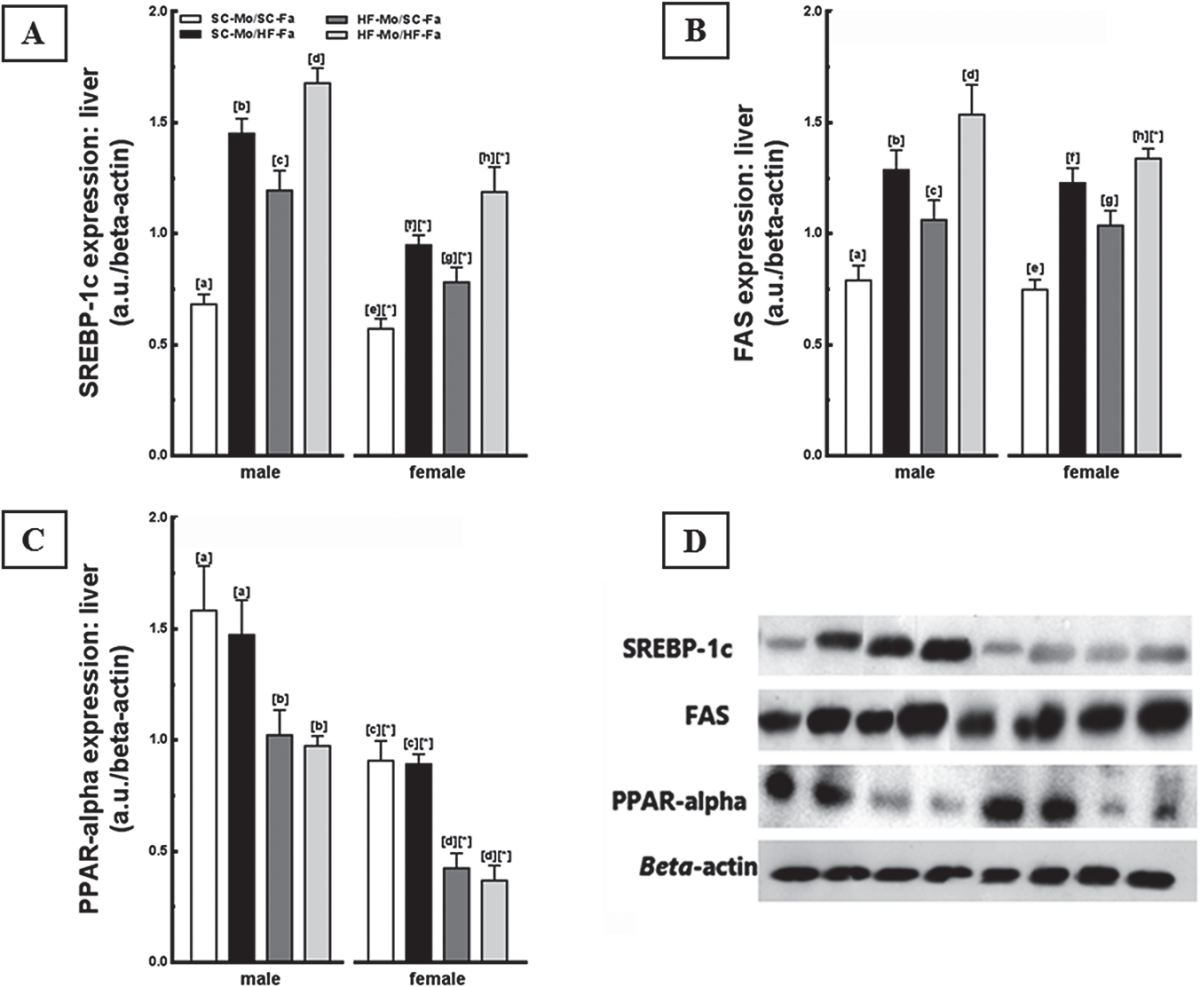
Molecular analyses in the offspring liver (panel 3). Liver immunoblotting corrected by beta-actin expression of the male and female offspring at 12-weeks old. (A) SREBP-1c, (B) FAS, (C) PPAR-alpha, and (D) representative immunoblotting with bands (expressed in arbitrary units, a. u.). Data are expressed as the mean and SD (N = 5 mice per group, one-way ANOVA and the posthoc test of Holm–Sidak). Same letters represent equal groups with no statistical difference while different letters represent different groups from each other, with statistical difference (*P*<0.05), * different from the corresponding counterpart (*P*<0.05). Abbreviations: sterol regulating element binding protein (SREBP-1c); fatty acid synthase (FAS); peroxisome proliferator activator receptor alpha (PPAR-alpha); offspring of SC mother and SC father (SC-Mo/SC-Fa); offspring of SC mother and HF father (SC-Mo/HF-Fa); offspring of HF mother and SC father (HF-Mo/SC-Fa); offspring of HF mother and HF father (HF-Mo/HF-Fa).

The findings obtained by gene expression were complemented with the analysis of protein expression. Male and female HF-Mo/HF-Fa offspring showed the highest values of SREBP-1c and FAS, especially males, reflecting an interaction between paternal diet, maternal diet, and offspring sex (*P*<0.001; three-way ANOVA, Fig 7). Inversely, a decreased expression of PPAR-alpha was observed in this offspring (*P*<0.001, Fig 7), equating to the offspring of HF-mothers.

The SC-Mo/HF-Fa offspring had higher SREBP-1c and FAS protein expression compared with SC-Mo/SC-Fa offspring (*P*<0.0001, Fig 7). Interestingly, the SC-Mo/HF-Fa offspring also presented increased SREBP-1c and FAS (+21% in both proteins and both sexes; *P* = 0.01) in relation to HF-Mo/SC-Fa offspring (Fig 7). However, HF-father had no influence on the protein expression of PPAR-alpha in male and female offspring. A decrease in these proteins was observed in HF-Mo/SC-Fa and HF-Mo/HF-Fa offspring (Fig 7).

Finally, the HF-Mo/SC-Fa offspring had elevation in protein expression of SREBP-1c and FAS and decrease in PPAR-alpha, compared to SC-Mo/SC-Fa offspring (*P*<0.001, Fig 7).

The two-way ANOVA test showed that the paternal diet or maternal diet and offspring sex favored the elevation in SREBP-1c, mainly in males (*P*<0.001; two-way ANOVA).

## Discussion

Maternal and/or paternal obesity induced by high-fat diet during preconception until lactation led to variations in BM, metabolic abnormalities, and alterations in liver structure due to changes in components of the beta-oxidation and/or lipogenesis pathways in male and female offspring at 12-weeks old. Interestingly, when only the father received the HF diet, the male and female offspring presented high fasting glucose, decreased glucose tolerance and liver steatosis. When only the mother received the HF diet, the offspring has more significant BM gain, exemplified by an increase in the adiposity index.

The parents in this study received a diet rich in lipids (49% of total energy), in which 36% of these stemmed from saturated fat from lard. The type of lipid can determine different phenotypes in the individual and may be worse than the obesity itself [20]. The HF diet formulation in the present study, had soybean oil combined with lard to avoid bias due to the absence of essential fatty acids (found in the soybean oil, but not in lard), as recommended by AIN-93G [15]. Currently, we know that a diet rich in saturated fatty acids might lead to changes in the action of insulin, with hyperglycemia, increased body mass, and a systemic proinflammatory state [20–21], which can be transmitted to other generations. As maternal diet modifies the development of fetal adipose tissue during pregnancy and lactation [22], it is possible to explain the increased body fat observed in the offspring. Consequently, with an increase in adipose tissue, there is a greater release of pro-inflammatory cytokines and decreased adiponectin, which will favor glucose intolerance and disorders in insulin sensitivity resulting in hyperinsulinemia, findings shown in this study that have been previously demonstrated [23].

Defects in insulin secretion and insulin action lead to multiple metabolic changes, such as hyperglycemia, increased hepatic glucose production through the overexpression of PEPCK and G6Pase, dyslipidemia and hepatic alterations in both sexes offspring of obese mothers, results corroborated in other studies [24–25]. Animals with overweight induced by maternal-fetal programming develop insulin resistance, which has distinct effects on adipose tissue and the liver. At first, it stimulates lipolysis, resulting in increased transport of FFAs to the liver by the portal vein and increased "input". Simultaneously, in the liver, hyperinsulinemia inhibits beta-oxidation reducing the "output"[26].

Consequently, maternal obesity induced by HF diet resulted in decreased gene and protein expression of peroxisome proliferation activator receptor (PPAR)-alpha and also decreased the transcription of genes involved in the formation of enzyme-linked lipid oxidation [27] in the mitochondria of offspring and is inversely correlated with the degree of liver steatosis. In addition, the decrease in CPT-1, a target gene of PPAR-alpha, reduced beta-oxidation by decreasing the transport of fatty acids into the mitochondrial matrix [28]. Concomitantly, SREBP-1c also augmented in the offspring, favoring lipogenesis due to the synthesis of fatty acids by FAS enzyme, which culminated in the accumulation of triacylglycerol in hepatic tissue [29–30]. According to our data, diet-induced maternal obesity led to an increase in SREBP-1c and FAS and a decrease in PPAR-alpha and CPT-1 demonstrating that when the mother received HF diet, the hepatic pathways are altered with impaired beta-oxidation and enhanced lipogenesis in adult offspring. These findings are corroborated by hepatomegaly and elevated hepatic triacylglycerol content.

Recent studies in animals and humans have demonstrated the paternal participation in postnatal life of the offspring [31–32]. Several theories have been proposed to elucidate how the nutritional status of the fathers can affect future generations, even if the father's involvement in the formation of the fetus is only in the act of fertilization and quite different from the mother who offers an intrauterine environment for the development of offspring. Paternal obesity induced by HF diet can program the descendants through delayed embryonic development [33] and epigenetic factors, resulting from a modification in the germ cells caused by environmental experience such as diet in the preconception period [34].

Wide varieties of paternal dietary models can induce damage in the offspring. In this context, a paternal low protein diet affected adult offspring cardiovascular and metabolic functions in male and female mice [35]. It is unlikely that chronic high-fat diet in fathers was able to induce impaired glucose tolerance and insulin secretion in adult female rat offspring [11]. These controversial results demonstrate that the type of paternal diet and the animal species used in the study directly influence the obtained findings. As such, as opposed to recent literature [11], we found that both sexes offspring of both diet-induced obese parents (mother and/or father) are affected by fetal programming, being the males generally more sensitive to parental insult, showing a sexual dimorphism. An important explanation for this is the use of the C57BL/6 strain, which emerged as a model for the study of metabolic alterations in rodents seeking comparisons with humans. These animals have a genetic vulnerability and are strongly influenced by environmental factors in the development of obesity and insulin resistance [36–37].

Our data are in accordance with the literature [11] and epidemiological studies that showed that paternal BM index has not been associated with infant birth size, in contrast to an obese mother [38–40]. However, paternal obesity induced by HF diet was able to alter carbohydrate metabolism, culminating in hyperglycemia, compensatory hyperinsulinemia, and subsequent glucose intolerance, effects independent of variations in BM, adiposity index and food intake, as previously demonstrated [11–12,41–42]. In addition, the decrease in adiponectin levels in these groups is associated with the results above because adiponectin exhibits insulin-sensitizing characteristics by inhibiting hepatic glucose production through inhibition of PEPCK and G6Pase and improving glucose homeostasis [43]. However, changes in insulin action can stimulate hepatic gluconeogenesis through the overexpression of key enzymes of such as PEPCK and G6Pase [44], also observed in offspring of the HF-father.

In the present study, we found that the HF-mother affects insulin levels and glucose tolerance of the offspring more intensely than when only the father received the HF diet. Several studies have shown that the offspring of obese mothers, fed with the HF diet are influenced by an adverse intrauterine environment and by the time of lactation in which breast milk is the vehicle for the nourishment of offspring [6,45]. Consequently, in addition to epigenetic alterations, the offspring generated by HF-mother also suffer direct interference from these factors, which could have resulted in greater prejudice in carbohydrate metabolism [46].

**Fig 8:**
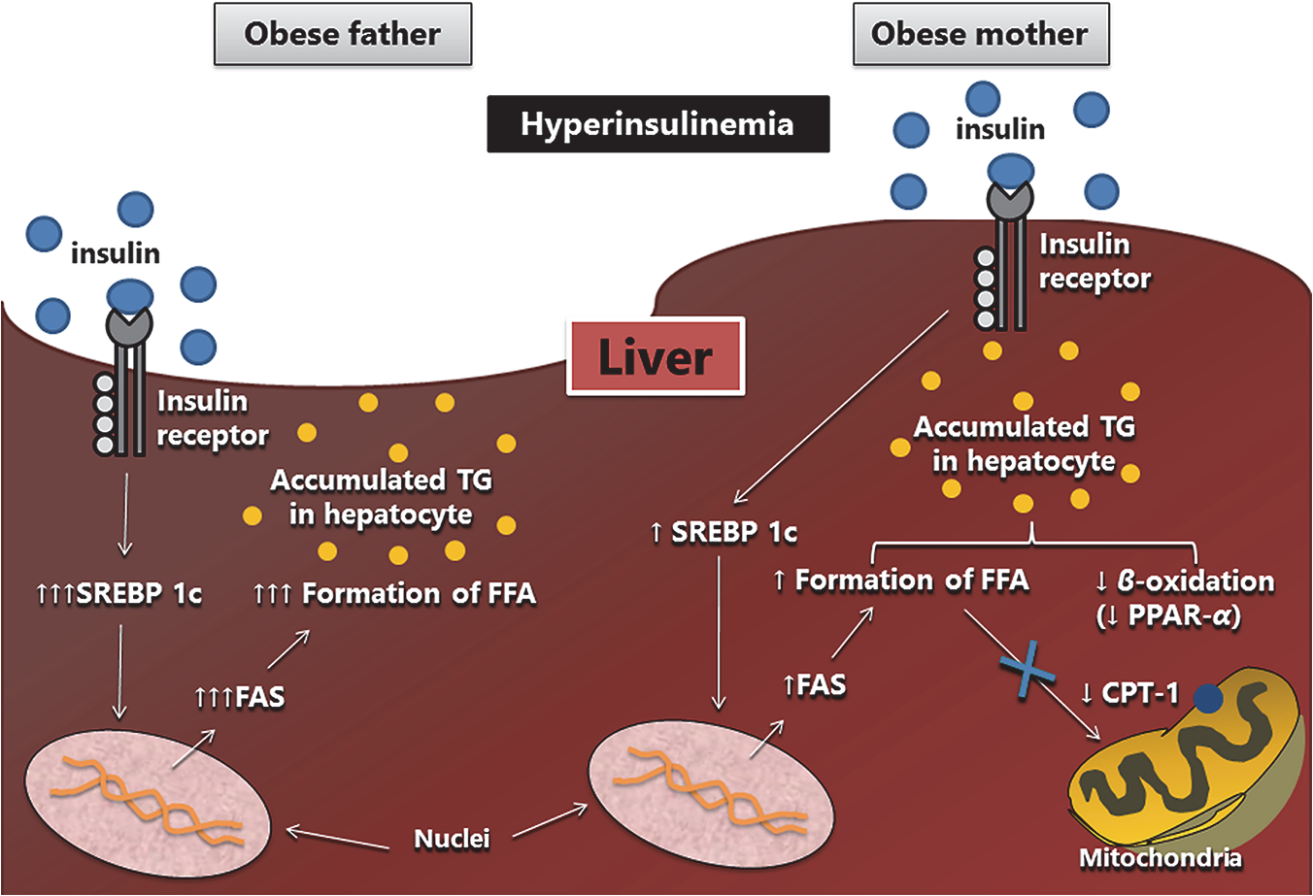
Scheme of the mechanisms obtained in the study. Liver steatosis observed in offspring of obese father and obese mother is possibly the result of different activated pathways, resulting from a framework of hyperinsulinemia. It is suggested that the paternal obesity intensifies the lipogenesis pathway through increased gene and protein expression of SREBP-1c, which stimulates the synthesis of fatty acids by the FAS enzyme. Maternal obesity led to a less intense lipogenesis and decreased beta-oxidation showed by a lower gene and protein expression of PPAR alpha and CPT-1. Arrow-up (↑) indicates an increase and arrow-down (↓) indicates a decrease in both gene and protein expressions. Abbreviations: sterol regulating element binding protein (SREBP-1c); fatty acid synthase (FAS); peroxisome proliferator activator receptor alpha (PPAR-alpha); carnitine palmitoyltransferase I (CPT-1); free fatty acid (FFA).

Interestingly, paternal obesity induced by the HF diet resulted in liver steatosis in male and female offspring, similar to programming resulting from maternal obesity. Meanwhile, our data suggest that the fat accumulation in the liver is due to different signaling mechanisms (see Fig 8). When only the father received the HF diet, liver steatosis would be caused by exacerbated lipogenesis, with significant increases in SREBP-1c and FAS enzyme (higher than that found in offspring of obese mothers) and unchanged beta-oxidation. Given the above, we hypothesized that one explanation for these different forms of liver steatosis programming is mitochondrial dysfunction. It is known that liver mitochondria are of maternal origin and, as such, may be an important target for the investigation of metabolic disorders in the offspring of HF-mother, corroborated by the fact that they constitute a critical site of energy metabolism [5]. The liver mitochondrial dysfunction may be associated with the development of non-alcoholic liver steatosis in obese mice due to the reduction in beta-oxidation of fatty acids, as occurred in male and female offspring ofHF-mother [47]. Additionally, the presence of liver steatosis in offspring of HF-father is supported by increased hepatic triglyceride content and hepatomegaly. We also believe that in the long term, the rise in the activation of lipogenesis, resulting from paternal programming, can damage the beta-oxidation pathway because the formation of fatty acids leads to higher intracellular concentrations of malonyl coenzyme A, which can inhibit the action of CPT-1 and thus impair beta-oxidation [48].

The male and female offspring of diet-induced obese parents had maximized deleterious effects in all observed variables. Interestingly, the pups of both HF-mother and HF-father had higher BM at birth, demonstrating an early influence of this type of fetal programming. A relevant study that examined the risk for type 2 diabetes and glucose intolerance in offspring of diabetic parents showed that the risk for type 2 diabetes among offspring with a single diabetic progenitor was lower than the risk when both parents are diabetic. These data suggest the simple additive model for parental transmission of diabetes, in which risk, when the mother and father are affected, is a function of the sum of risk when either parent is diabetic [14], a fact that underscores our results.

Studies have already defined that any changes noted in offspring of diet-induced obese father may be phenotypically expressed if it happens in conjunction with maternal factors (ecological, physiological or molecular). This implies that the transmission between generations may occur [34], configuring another hypothesis that could explain how the offspring of both diet-induced obese parents had a worse metabolic profile and liver changes, even though the HF-father alone does not determine these variables. Thus, the presence of glucose intolerance and hyperinsulinemia in the offspring ofHF-father may indicate early damage to other systems, which become intensely concerned when there is a maternal insult (in the case of this study, the diet-induced obesity during the preconception period). Likewise, the maximized steatosis in the male and female offspring generated by both diet-induced obese parents suggests that hepatic lipogenesis and beta-oxidation pathways are affected because of the sum of the isolated effects from the HF-mother and HF-father.

Finally, other studies should be conducted to infer the mechanisms involved in maternal and paternal programming in several tissues and systemic metabolism. The data obtained in the present study suggest that the paternal obesity induced by HF diet can result in alterations in the metabolism of carbohydrates, activating components of lipogenesis in the liver and leading to liver steatosis. All these effects are independent of BM. In contrast, maternal obesity induced by HF diet results in changes in metabolic profile and liver resulting from activation of hepatic lipogenesis with impaired beta-oxidation; these effects are related to augmented BM. When both parents received a HF diet, the effects observed in the male and female offspring are compounded.
